# Nano-FTIR chemical mapping of minerals in biological materials

**DOI:** 10.3762/bjnano.3.35

**Published:** 2012-04-05

**Authors:** Sergiu Amarie, Paul Zaslansky, Yusuke Kajihara, Erika Griesshaber, Wolfgang W Schmahl, Fritz Keilmann

**Affiliations:** 1 Max Planck Institute of Quantum Optics and Center for NanoScience, 85748 Garching, Germany; 2 Max Planck Institute of Colloids and Interfaces, Wissenschaftspark Golm, 14424 Potsdam, Germany; 3 Department of Basic Science, The University of Tokyo, Tokyo 153-8902, Japan; 4 GeoBio-Center at LMU and Department of Earth and Environmental Sciences, Ludwig-Maximilians-Universität, 80333 München, Germany

**Keywords:** biomineralization, chemical mapping, infrared spectroscopy, nanocrystals, optical near-field microscopy

## Abstract

Methods for imaging of nanocomposites based on X-ray, electron, tunneling or force microscopy provide information about the shapes of nanoparticles; however, all of these methods fail on chemical recognition. Neither do they allow local identification of mineral type. We demonstrate that infrared near-field microscopy solves these requirements at 20 nm spatial resolution, highlighting, in its first application to natural nanostructures, the mineral particles in shell and bone. "Nano-FTIR" spectral images result from Fourier-transform infrared (FTIR) spectroscopy combined with scattering scanning near-field optical microscopy (s-SNOM). On polished sections of *Mytilus edulis* shells we observe a reproducible vibrational (phonon) resonance within all biocalcite microcrystals, and distinctly different spectra on bioaragonite. Surprisingly, we discover sparse, previously unknown, 20 nm thin nanoparticles with distinctly different spectra that are characteristic of crystalline phosphate. Multicomponent phosphate bands are observed on human tooth sections. These spectra vary characteristically near tubuli in dentin, proving a chemical or structural variation of the apatite nanocrystals. The infrared band strength correlates with the mineral density determined by electron microscopy. Since nano-FTIR sensitively responds to structural disorder it is well suited for the study of biomineral formation and aging. Generally, nano-FTIR is suitable for the analysis and identification of composite materials in any discipline, from testing during nanofabrication to even the clinical investigation of osteopathies.

## Introduction

**Figure 1:**
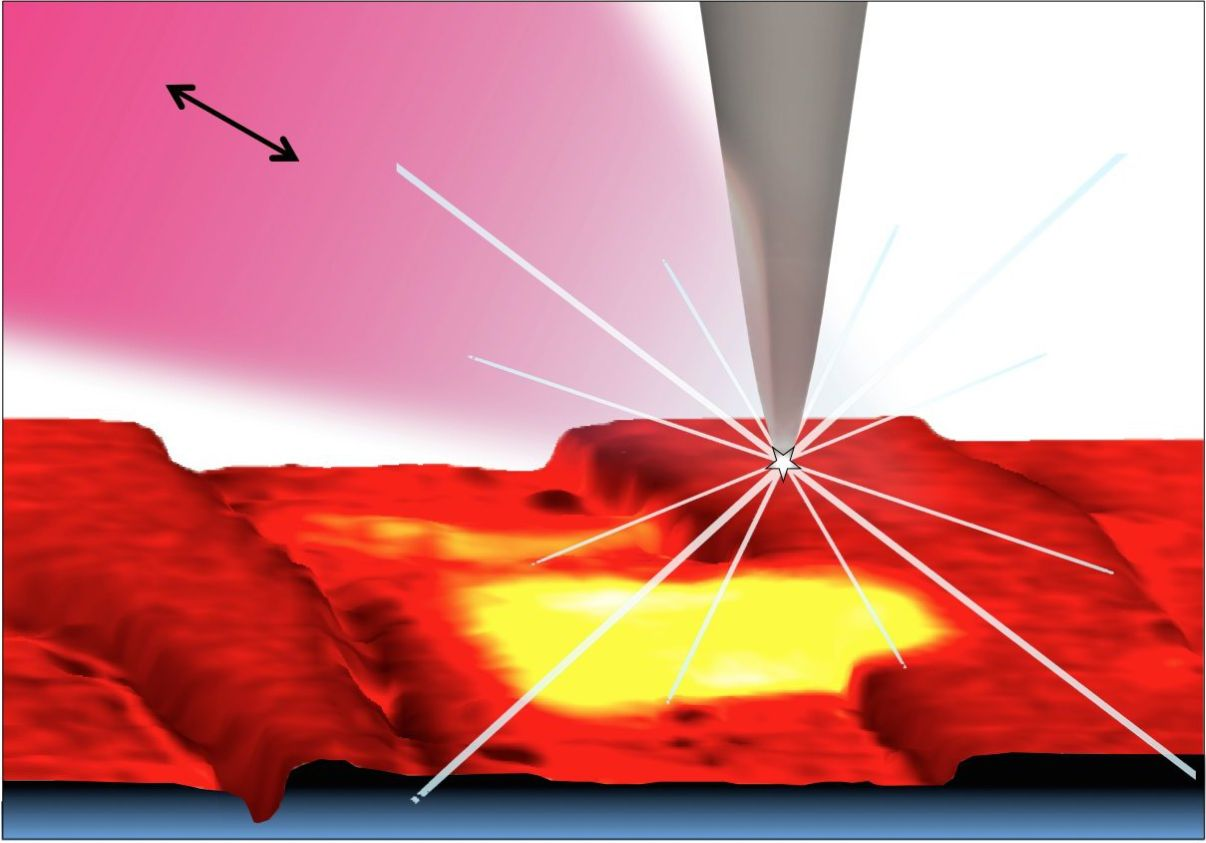
Nano-FTIR basic interaction. Focused infrared light incident from the upper left excites a nanofocus at the metal tip, symbolized as a star, which interacts with the scanning sample. The backscattered infrared light carries local information. Here, the infrared response (color code as in Figure 2b) is overlaid on a pseudo-3D rendering of the topography, which is simultaneously recorded, of a 1.4 × 1.6 µm^2^ zoom area designated in Figure 2b by the dashed box on the left. Topographic height differences ca. 50 nm.

Fourier-transform infrared spectroscopy (FTIR) [1] is a standard tool in chemical analysis. It can identify virtually any substance through the "fingerprint" of the molecular vibrational absorption spectrum in the 3–30 µm wavelength region. Nano-FTIR spectroscopic near-field microscopy is a fascinating recent advance [2–4]. It enables scattering near-field optical microscopes (s-SNOM) [5–6] to operate at ultrahigh spatial resolution over a broad mid-infrared spectrum emitted from either a coherent supercontinuum source [2–3] or an incoherent thermal source [4]. The s-SNOM uses a metalized AFM tip as a light-concentrating antenna such that the sample is probed with a nanofocused light field (Figure 1). The nanofocus is a light spot of the same size as the tip radius, which thus defines both the optical and the topographic resolutions of s-SNOM. Detection of the backscattered light reveals local optical information. The probed volume extends typically 20 nm laterally, as well as into the sample (sometimes even less than 10 nm) [7]. The high resolution is independent of the wavelength. This enables the utilization of long wavelengths corresponding to the infrared fingerprint vibrations. s-SNOM has been successfully operated with visible, infrared and terahertz illumination, and has been applied to organic [8–9] and inorganic [10] materials, in such diverse fields as nanoelectronics [11], the physics of phase transitions [12], or material identification [13]. The underlying near-field interaction has been theoretically modeled and experimentally verified. The observable contrasts and spectra can be derived from the complex dielectric function of the sample material [6,14], and include both the absolute efficiency and the phase of the scattering [3]. Nano-FTIR has, up to now, been demonstrated with flat test samples only, consisting of metals, semiconductors and polar crystals [3–4].

Hard biological tissues are highly textured composites of submicrometric inorganic particles embedded in organic matrices [15–17]. Major tissues of interest include the phosphatic (bone) family of materials, and the carbonatic family as found, e.g., in mollusc shells. Within the phylum Brachiopoda, both strategies of hybrid shell architecture have evolved: Calcium carbonate crystals in an organic matrix [18–20], and laminates of calcium phosphate nanoparticle reinforced chitin fibers [21–22]. FTIR spectroscopic microscopy is a well-established method and has been extensively used to study bone biominerals at several micrometers spatial resolution [23–30]. Its strength in the study of bone biopsies, mineralized tendons, dentin or ivory is mainly due to a broad absorption band between 950 and 1150 cm^−1^ assigned to the ν_3_ vibration of the PO_4_^3−^ ion of apatite. It is thus possible to acquire maps of mineral concentration, and to relate mineral to protein (collagen) or carbonate distributions, usually revealing considerable spatial variation. Moreover, the apatite band exhibits a weak spectral substructure, evident from Fourier self-deconvolution [1]. It reveals relative weights of apatite species that are assigned, with the help of chemical and X-ray analyses, to Mg^2+^, F^−^ or CO_3_^2−^ substitution, or differing particle size, or crystal imperfections [24,31–32]. In this study we demonstrate the power of nano-FTIR to map naturally formed mineralized nanostructures. We show that we obtain fingerprint information on two example systems of biominerals. Experimental comparison is made with electron microscopy (SEM) to verify that nano-FTIR perfectly matches what is already known about the structures, and that the method indeed provides rich chemical and structural contrasts at the 20 nm scale.

## Results

### Marine shell as an example of a carbonate-forming organism

**Figure 2:**
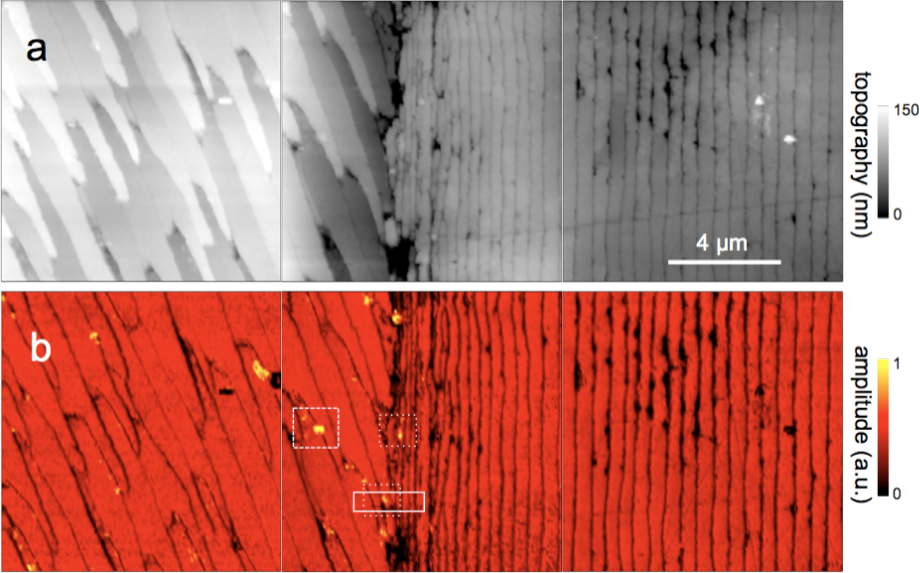
Polished section of *Mytilus edulis* viewed in monochromatic s-SNOM (980 cm^−1^). (a) Topography of the interface between two calcium carbonate polymorphs, biocalcite crystals (left) and bioaragonite crystals (right); (b) the backscattered infrared amplitude (*n* = 3) contrasts the organic matrix at a relatively low level; a few, unexpected particles highlighted by their enhanced amplitude are chemically different; we refer to them as "phosphate" crystals because their spectra (Figure 3 and Figure 4) are characteristic of phosphate.

We demonstrate nano-FTIR near-field microscopy on a *Mytilus edulis* (*M. edulis*) shell specimen, which exhibits easily resolvable fibrous biocalcite microcrystals and tablet-shaped bioaragonite nanocrystals. On a polished section, the expected characteristic interface [33] between an outer calcite layer and an inner aragonite layer is readily located with the help of an overview microscope (0.7 µm resolution) built into the commercial s-SNOM used (neaspec.com). On the inner side there is an interlayer, ca. 2 µm wide, with modified bioaragonite crystals [34]. Three adjoining s-SNOM images of 10 × 10 µm^2^ area each were consecutively acquired and stitched together. The topography (Figure 2a) exhibits the arrangement of (i) fiber-shaped biocalcite crystals with slightly oblique, flat surfaces at a few distinctly different heights, (ii) deep depressions mainly in the interlayer, and (iii) bioaragonite crystals with flat surfaces at equal height, some (in the interlayer) as narrow as 100 nm.

**Figure 3:**
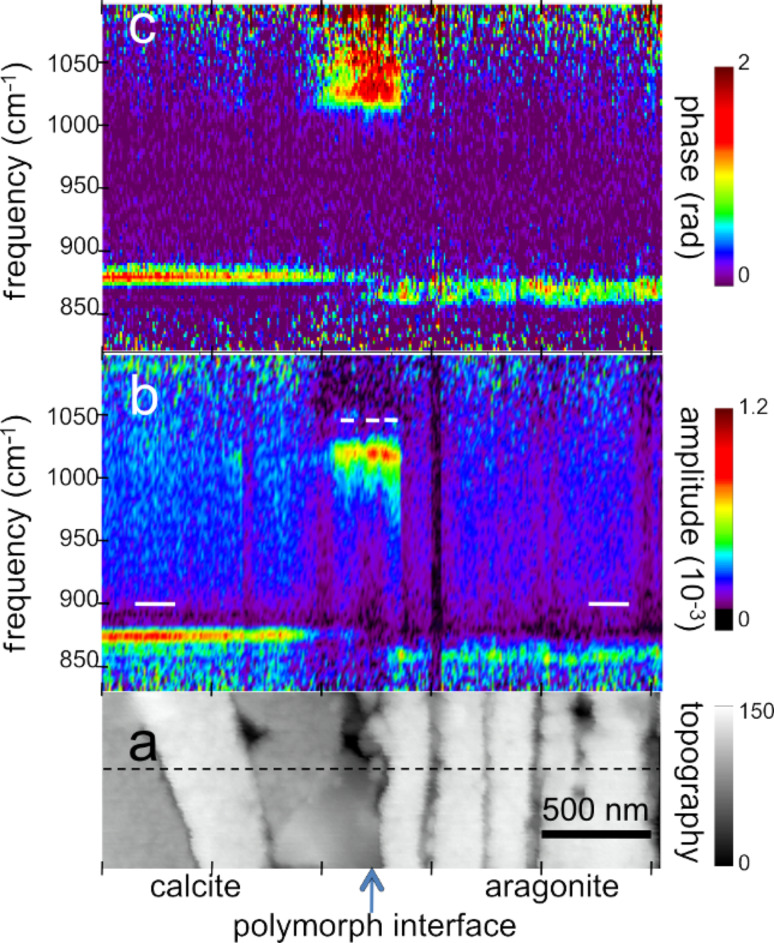
Nano-FTIR spectral line scan across the interface between calcite and aragonite layers; (a) Topography corresponding to the full white box of Figure 2b, with the scan line marked by a dashed line; (b) infrared amplitude and (c) infrared phase spectra identify calcite, "phosphate" and aragonite by their resonances at 872, 1018, and 857 cm^−1^, respectively. The white bars define the ranges of averaging for the spectra for Figure 4.

**Figure 4:**
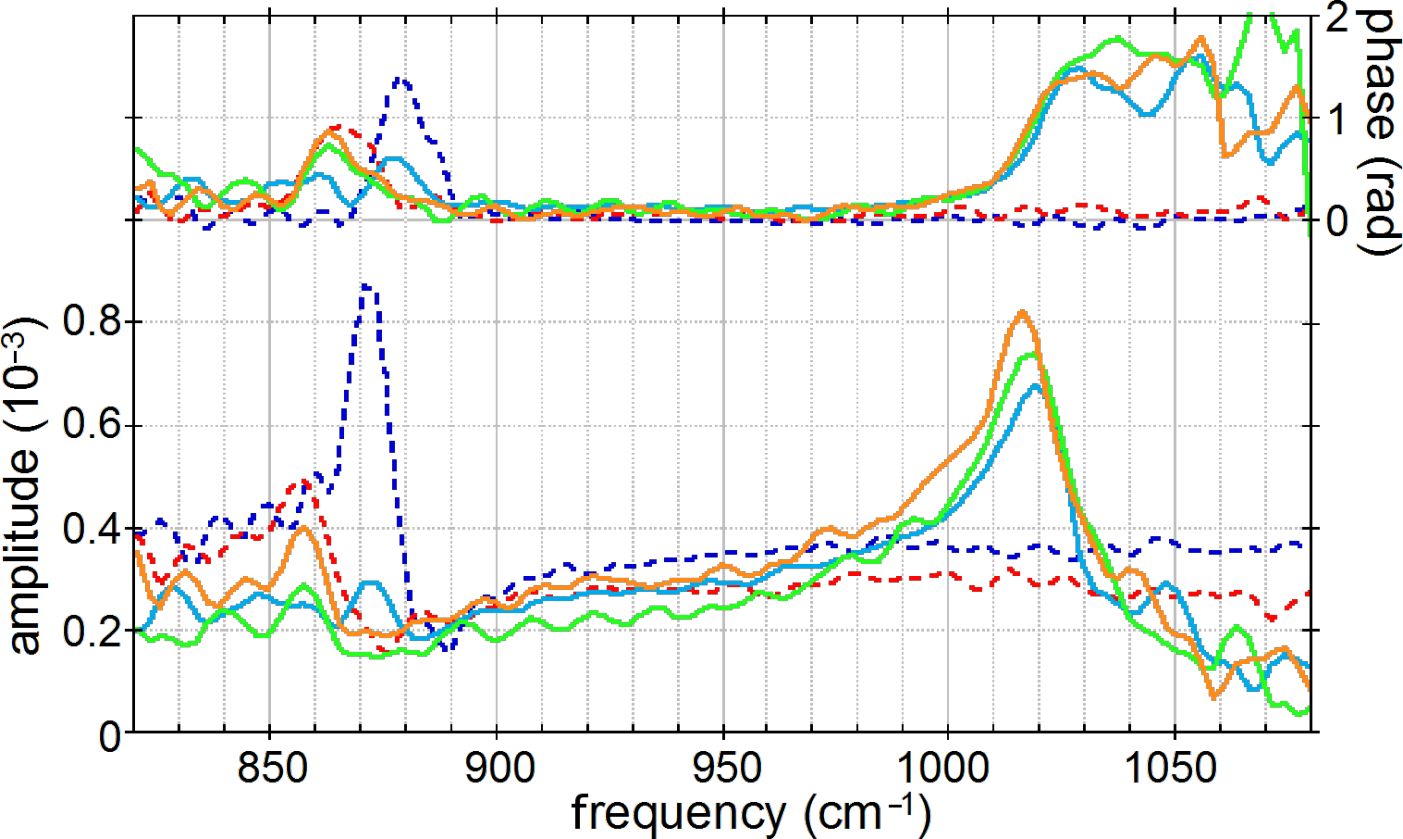
Nano-FTIR spectra of *M. edulis* obtained from Figure 3b and Figure 3c by averaging over the ranges indicated in Figure 3b by the white bars: calcite region (blue, dashed), aragonite region (red, dashed), "phosphate"/interface region (full, from left to right: yellow, green, blue).

**Figure 5:**
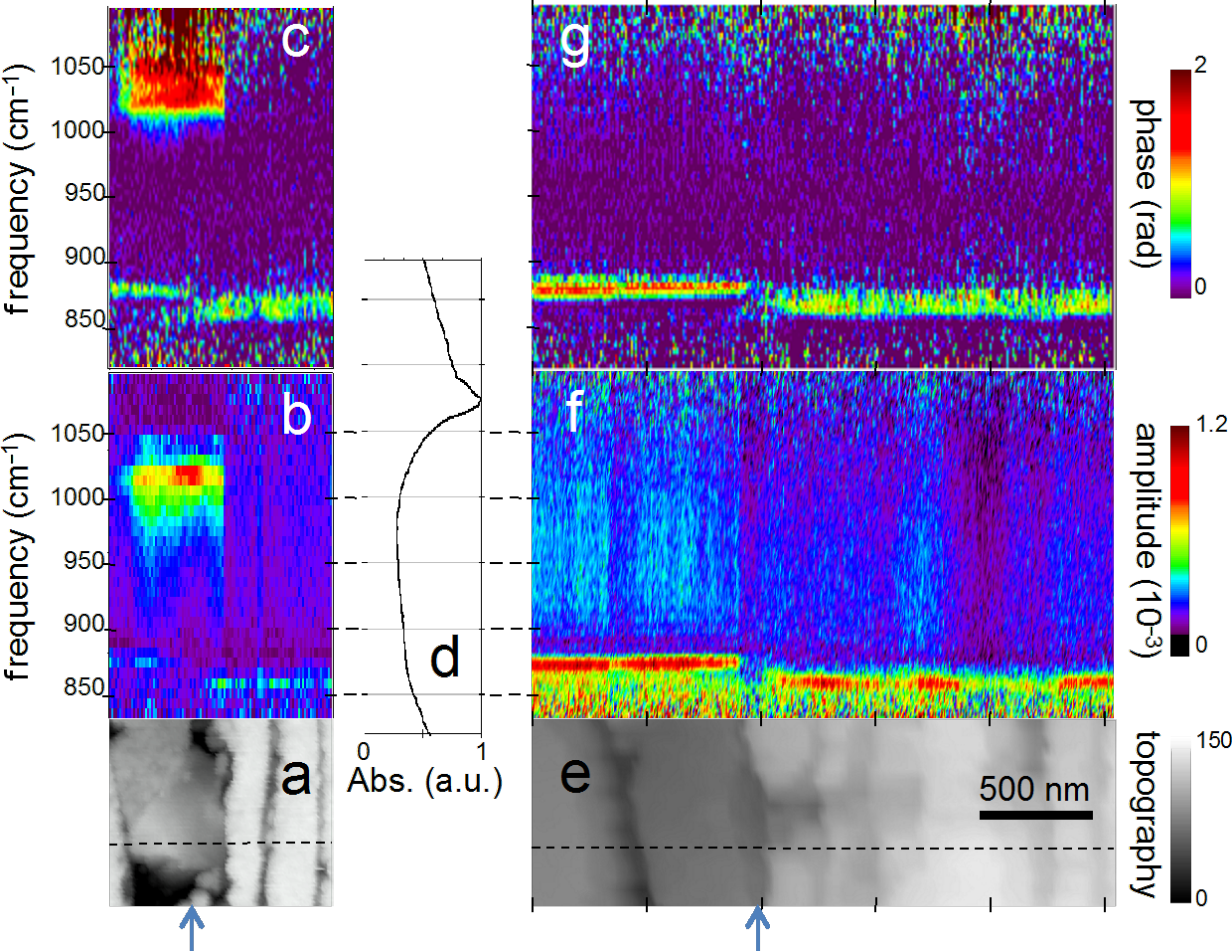
Nano-FTIR spectral line scans as in Figure 3; (a–c) Topography, amplitude and phase spectra ca. 300 nm below the scan in Figure 3; (e–g) the same but further away; (d) infrared absorption of dry polished material, with frequency scale aligned to (b and f).

For chemical mapping we collected 300 nano-FTIR spectra along a 2.5 µm line marked in Figure 3a, across the interface region designated by a full white rectangle in Figure 2b (Figure 3, additional scans are shown in Figure 5). The spectra in Figure 3b and Figure 3c (and also the extracted averaged spectral profiles in Figure 4) are dominated by a single, sharp resonance, which differs in frequency position for orthorhombic aragonite (855 cm^−1^) and trigonal calcite (873 cm^−1^), and thus both calcium carbonate polymorphs can be readily distinguished. The biocalcite spectra show no spectral shift within a given crystal, either upon comparison of neighboring crystals of the same type, or with changes in topographic height as seen with the three leftmost (biocalcite) crystals in Figure 3. Intriguingly, we notice on close inspection of all biocarbonate surfaces, e.g., in Figure 1, a shallow amplitude modulation on a 50–200 nm lateral scale, which we tentatively explain to be due to a mesocrystalline substructure that has been recently observed by SEM [35].

In Figure 3 and Figure 5 the infrared resonance is not as repeatable on the bioaragonite as on the biocalcite crystals, both with regard to spectral position and height. Further away from the interface layer bioaragonite has a more stable spectrum (not shown). This indicates that the interlayer carbonate (i) is truely bioaragonite but (ii) has a reduced, changeable mineral content. The interlayer bioaragonite crystals are clearly smaller and less well ordered (Figure 2).

**Figure 6:**
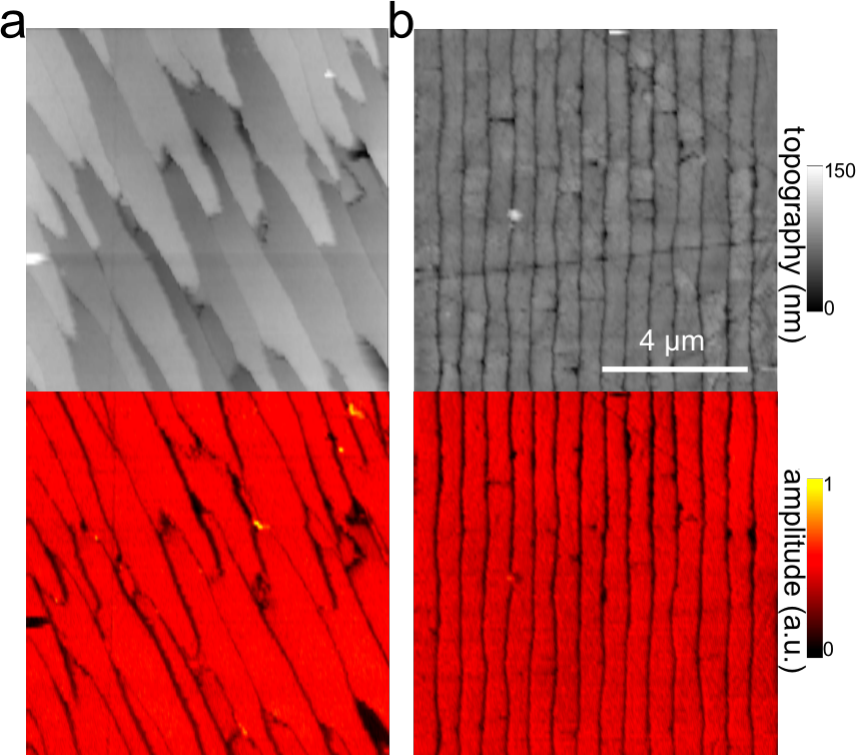
Polished section of *Mytilus edulis* viewed in monochromatic s-SNOM (980 cm^−1^), continued from Figure 2; (a) 120 µm further left in the calcite region and (b) 120 µm further right in the aragonite region.

Surprisingly, we find in Figure 3 a 350 nm long section with a similarly strong and sharp resonance at a much higher frequency of about 1018 cm^−1^, which we tentatively assign to be phosphate (see Discussion section). In order to specifically map its occurrence we acquired monochromatic s-SNOM images (Figure 2 and Figure 6) at 980 cm^−1^, which is a CO_2_ laser frequency at which the scattering signal is still weakly enhanced by the "phosphate" resonance (see amplitude spectra in Figure 3b, Figure 4 and Figure 5b). "Phosphate" occurs at a few spots only, in the calcite region up to and including the interlayer, but not further out in the aragonite region; in the calcite region its occurrence diminishes with distance from the interface (Figure 6). Additional zoomed images such as Figure 1 (see also Figure 11) unveil individual "phosphate" regions as sparsely scattered, contiguous particles of 100 to 500 nm size that seem to be no higher than 10 or 20 nm judging from their topography. All "phosphate" particles are distinguishable in the topography images. In passing we note that there are numerous other nanoparticles with a topographic height of up to 100 nm (Figure 2 and Figure 6); these exhibit a small infrared amplitude pointing to a nonresonant organic material [6]. The "phosphate" particles in Figure 3 and Figure 5a–c happen to bridge the interface as they exhibit in their left part a reduced calcite resonance (best recognizable in the phase spectra), and in their right part also a reduced aragonite resonance. This superposition of spectra suggests that the "phosphate" particles are thin enough, about 20 nm, for sensing also of the underlying calcite or aragonite (see Discussion section) [36].

### Human dentin

**Figure 7:**
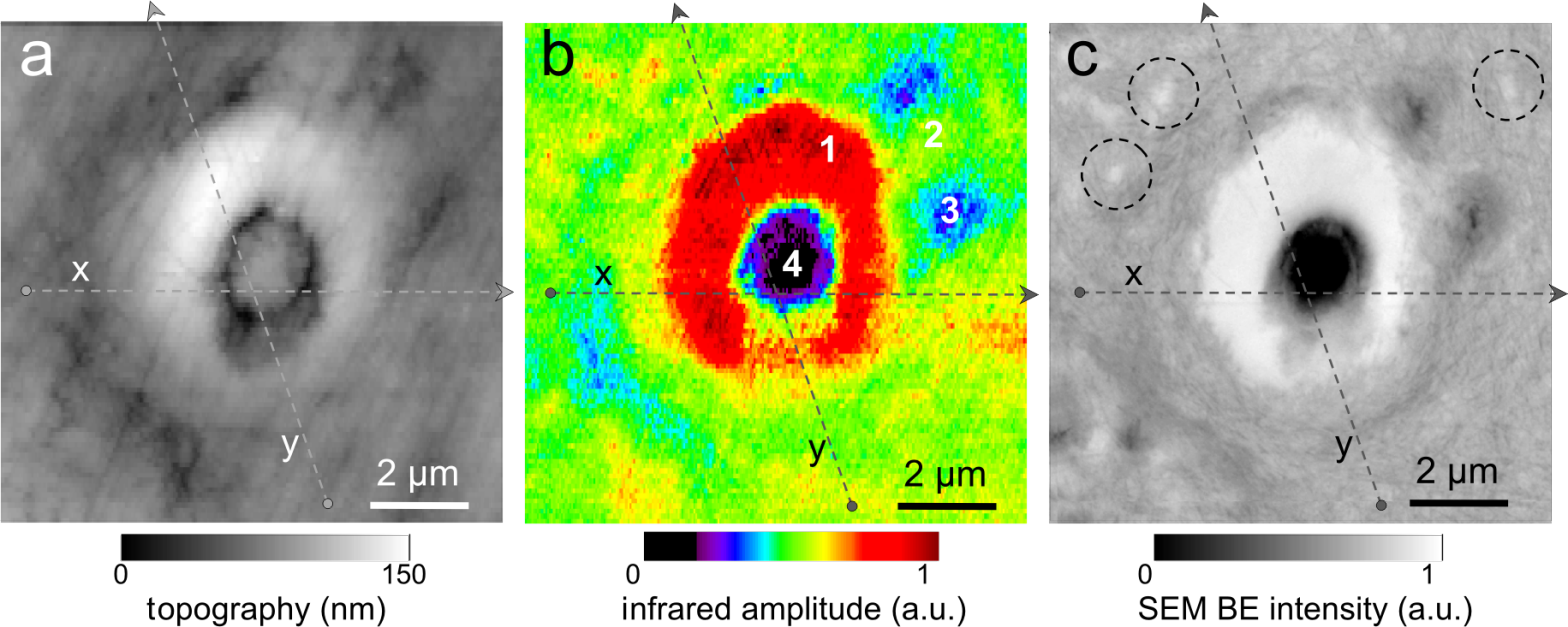
Tubule in human dentin and its surrounding imaged by (a,b) nano-FTIR where the topography (a) is acquired simultaneously with the infrared amplitude image (b, in spectrally integrating mode), and by (c) SEM (backscattered electron image (BEI)).

The exciting potential of nano-FTIR imaging of phosphate-based biominerals is demonstrated with a human tooth in which the bone-like phosphate nanocrystals are too small to be resolved, but where a hierarchy of structures exists at all scales including the submicrometer range [16,37]. Here, we explore dentine, a mineralized collagen-fibril-based [37] biological composite that supports enamel and is similar to bone, but does not remodel and does not contain cells. Dentin contains extensions of the pulp cells, which reside in ca. 1 µm thick tubules that are often surrounded by 1 to 2 µm thick mineralized sheaths devoid of collagen. Tubules of teeth are known to branch into nano-tubuli [38] and are easily observed on perpendicularly cut sections. Figure 7 shows a 10 × 10 µm^2^ area surrounding a typical tubule (filled with PMMA, see methods) inside the tooth, approximately 1.5 mm away from the enamel/dentin junction. The infrared image in Figure 7b was obtained in a spectrally integrating mode that highlights the phosphate band from approx. 950 to 1150 cm^−1^ (see Experimental section). A general similarity is seen with the SEM backscattered electron (BE) image (Figure 7c), in which the intensity is known to be a measure of the mineral density, averaged over ca. 1 µm depth [39]. The high-density peritubular rims produce the strongest infrared scattering. As a consequence of their higher resistance to polishing, they protrude approximately 50 nm from the surrounding dentin matrix. Outside the peritubular ring, the upper-right quadrant of the BE image exhibits two low-density spots with about 100 nm diameter designating nanotubuli. They appear clearly in the infrared images as spots of about 1 µm in diameter with an amplitude between the low one of PMMA (inside the large tubule) and that of intertubular dentin. Several high-density patches revealed by BEI (e.g., encircled in Figure 7c) are clearly expressed in the infrared image by a slightly enhanced amplitude. Thus spectrally integrated (phosphate band) nano-FTIR images of dentin compare well with the BE image of the same tissue.

**Figure 8:**
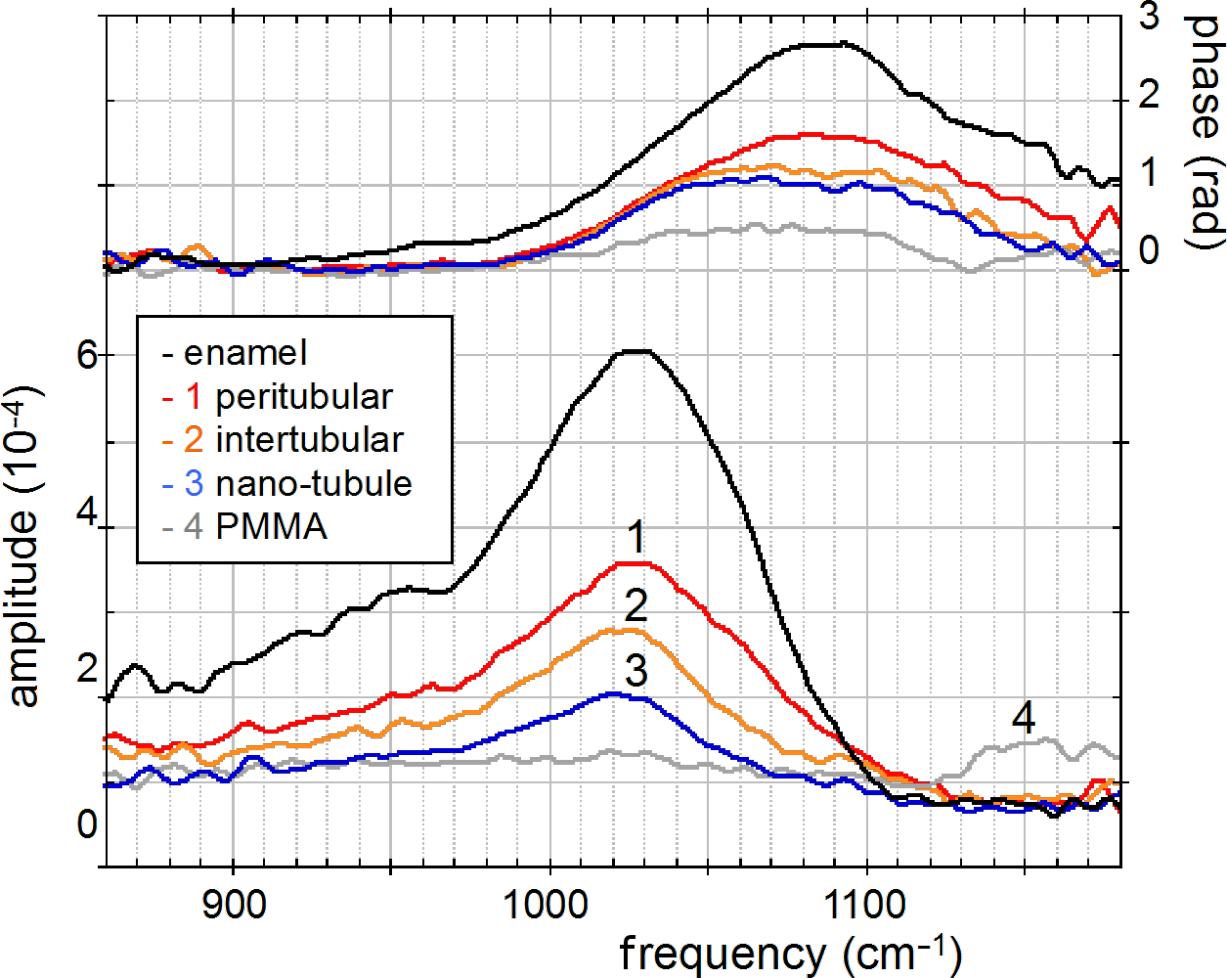
Local infrared spectra registered by nano-FTIR at positions 1–4 in Figure 7b (colors), and further away on enamel of the same tooth section (black).

The unique spectroscopic information provided by nano-FTIR is substantiated by four spectra centered on the phosphate ν_3_ vibrational band (Figure 8), acquired at characteristic locations marked in Figure 7b, and a fifth on the same tooth section taken 2 mm away on the enamel. The enamel and dentin amplitude (and phase) spectra are dominated by the resonance at 1025 cm^−1^. The peak height varies with location similarly as the spectrally integrated infrared amplitude as imaged in Figure 7b. While being assigned to phosphate as in the *M. edulis* case, we find that the tooth phosphate resonance is significantly broadened. Apparently this is an inhomogeneous broadening akin to what is known from classical FTIR spectra of bone [23–32]. Interestingly, the processed spectra show that the band shapes are significantly different for each of the five locations (see Discussion section, Figure 12a). The PMMA amplitude spectrum shows a relatively weak resonance around 1160 cm^−1^.

**Figure 9:**
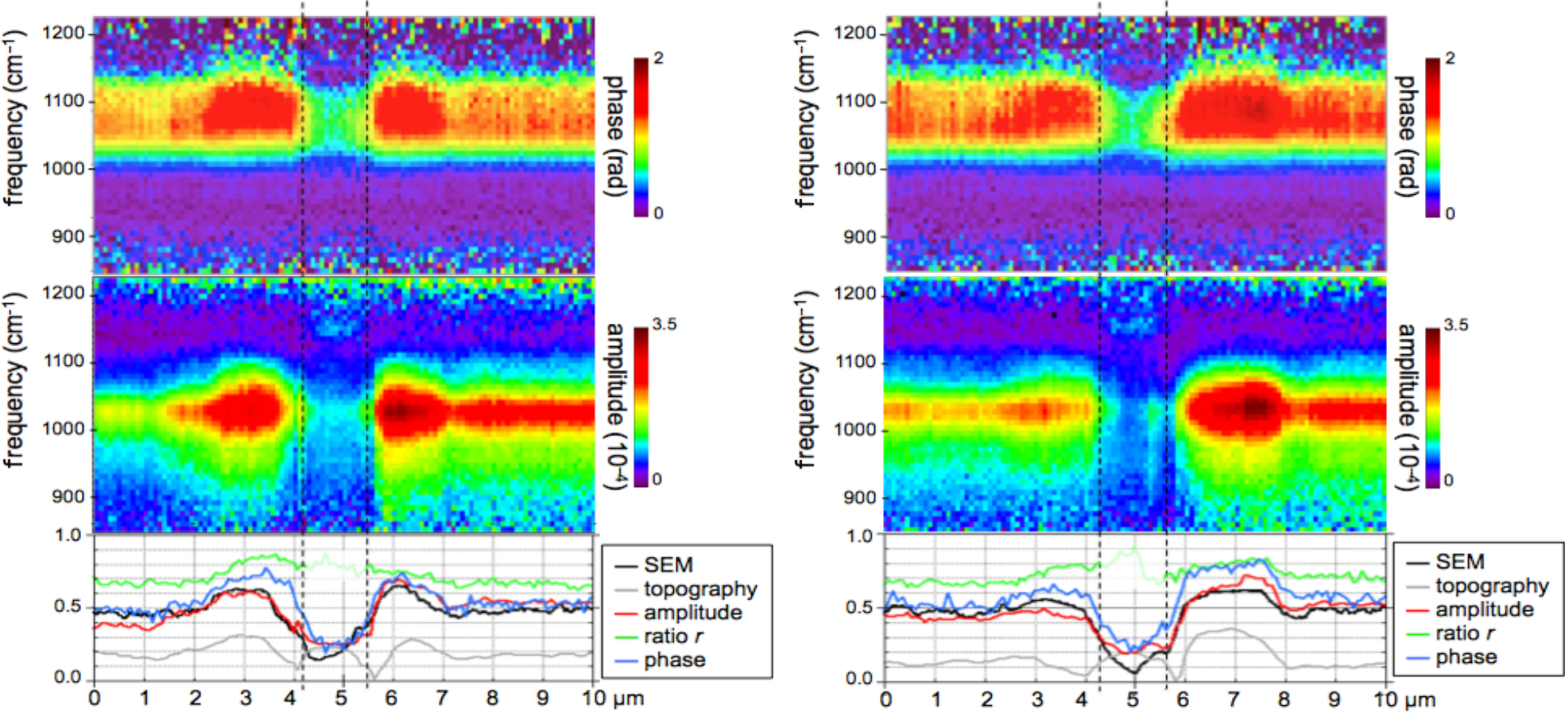
Dentin characteristics along line scans marked x (left part) and y (right part) in Figure 7; SEM intensity (black), topography (grey, in nm scaled by 1/240), nano-FTIR spectra (upper two panels), and three therefrom extracted quantities: amplitude at 1020 cm^−1^ (red, scaled ×2500), ratio *r* of amplitudes at 1053 and 1022 cm^−1^ (green), and phase at 1080 cm^−1^ (blue, in rad scaled by 1/2).

**Figure 10:**
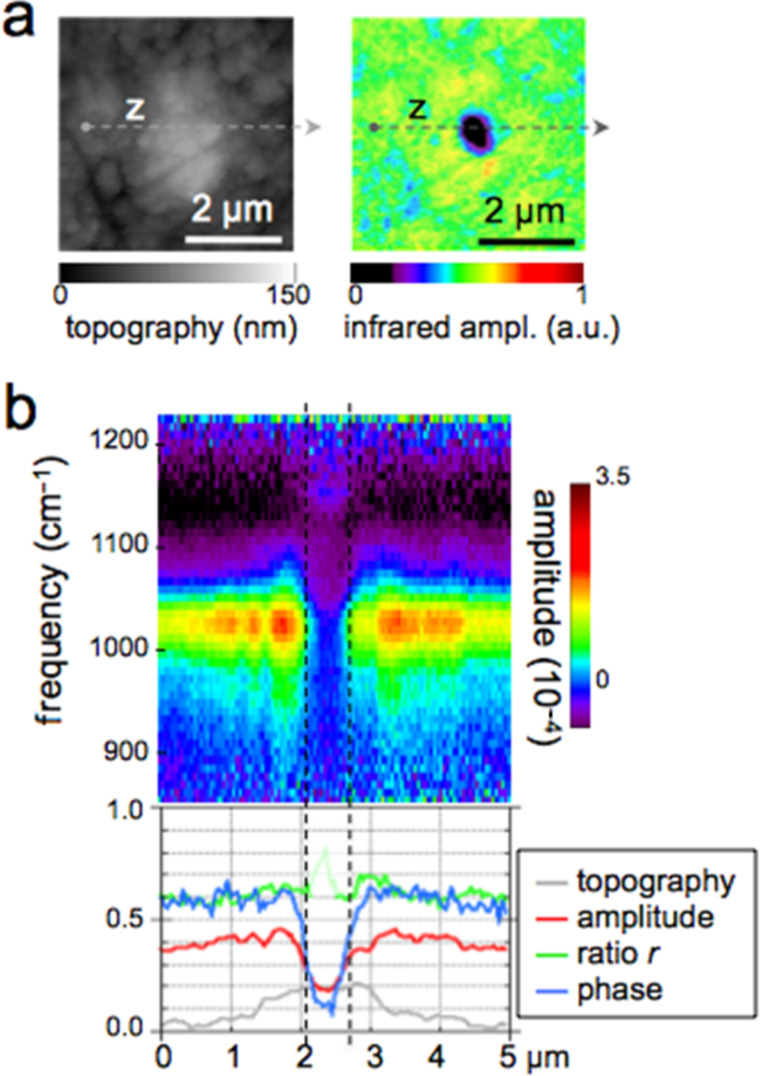
Tubule in human dentin near the enamel, imaged by nano-FTIR in which the infrared amplitude image (a, right, in spectrally integrating mode) is acquired simultaneously with the topography (a, left); (b) dentin characteristics along line scan marked z in (a), topography (grey, in nm scaled by 1/240), infrared amplitude spectrum (upper panel), and extracted quantities as in Figure 9.

The nano-FTIR spectra (Figure 9) recorded along 110 pixels of 10 µm long trajectories (marked in Figure 7) demonstrate the excellent reproducibility of the method. Local spectral variations clearly mark the dentin microstructure. Though weak, the spectrum of PMMA allow us to define the approximate tubule opening, designated by two dashed lines in Figure 9 and Figure 10. The lower panels of these figures show three interesting quantities extracted from the nano-FTIR spectral scans (colored curves, see Discussion section), together with the simultaneously recorded profiles of SEM intensity (black curve) and of topography (grey curve). A second tubule adjacent to the enamel junction displays a considerably narrower lumen and little topographic height, but clear infrared signatures (Figure 10).

## Discussion

FTIR is well established as a method for studying biominerals in a variety of settings, and nano-FTIR extends this functionality to include spectroscopic mapping at the nanometer length scale. Our observations of phosphates and carbonates in the well-studied examples of *M. edulis* and human dentin reveal exquisite detail, which matches what is observed by electron microscopy and nanoindentation. The achievement of chemical and structural mapping of biominerals opens new horizons for our understanding of mineral arrangements and variability in biological systems. Intricate carbonate-based natural skeletons, that may include transient and stabilized amorphous phases, can now be mapped within and across interfaces by a noncontact and nondestructive imaging technique. With respect to apatite studies, our method is directly applicable to the investigation of healthy and diseased forms of vertebrate bones and teeth. Mineral precipitation, aggregation and aging can now be analyzed and quantified in submicrometer detail, to better understand the biological processes of bone formation, abnormal development, and healing in response to drug treatment.

Several technical advantages of surface scanning make the nano-FTIR approach extremely robust and useful for the study of biological materials. The samples need not be thin, only reasonably flat, thus avoiding thin-section preparations, which are prone to damage. Unavoidable topographic obstacles resulting from the cutting and polishing procedures are of little consequence: Height variations of 100 nm do not change the off-resonant infrared amplitude (Figure 2) nor the resonant response in amplitude and phase, as demonstrated for example by the repeatability of the carbonate resonance spectra within the sample region containing biocalcite (Figure 3). At steep topographic edges though, the s-SNOM amplitude is known to be reduced over a width equal to the spatial resolution, resulting in "edge darkening" [6]. This effect probably contributes to the dark regions seen between calcite crystals in Figure 2b and remains to be further investigated. The impressive spatial resolution of nano-FTIR can be judged from the edges of the biocalcite crystals (Figure 3a) that demonstrate a mechanical (AFM) resolution certainly below 30 nm. Abrupt edges of the nano-FTIR line section showing the phosphate resonance (Figure 3b,c and Figure 5b,c) prove that the infrared resolution is better than 20 nm.

**Figure 11:**
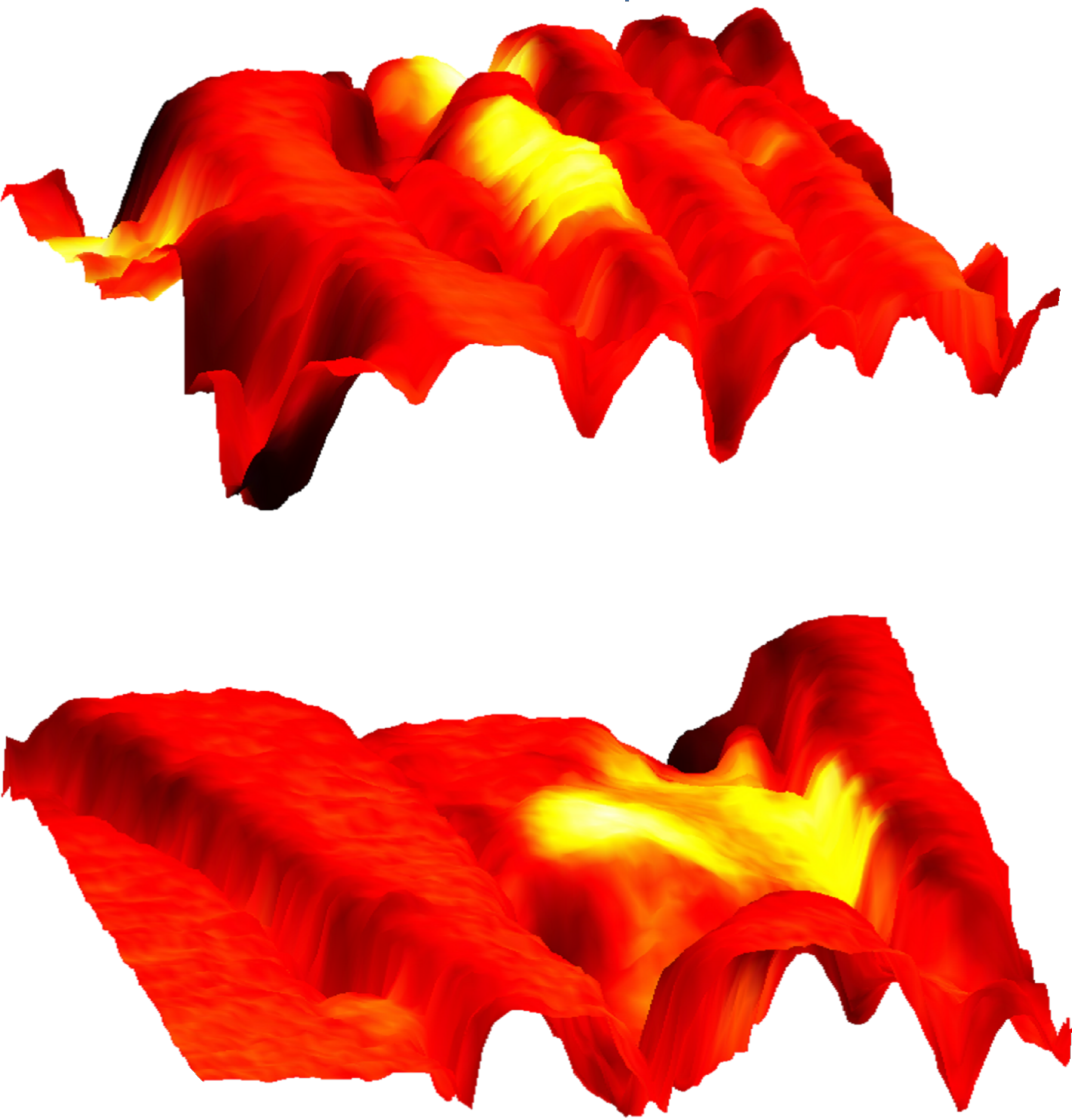
"Phosphate" in *M. edulis*. High-resolution images (similar to Figure 1) of the dotted-box areas, 1.0 × 1.2 µm^2^ of Figure 2. Backscattered infrared amplitude in color code as in Figure 2b, overlaid on a pseudo-3D rendering of the topography.

The "phosphate" particles in *M. edulis* are clearly recognized from their spectral signature (Figures 3–5), but would have been barely detected based on their topographic appearance alone (interestingly, their surfaces (see also Figure 1 and Figure 11) appear smoother than the neighboring biocarbonate crystals). Note that the nano-FTIR spectra of the "phosphate" particles additionally show one of the carbonate resonances (Figures 3–5), obviously originating from the crystals underneath [36,40]. To understand this effect, we recall that the basic near-field interaction probes the sample to a depth on the order of the tip radius (or somewhat deeper when one chooses the tapping amplitude or the average tip-to-sample distance to be larger than the tip radius) [6]. Thus buried objects may affect the backscattering provided that the covering layer is not thicker than a few times the tip radius [36]. Based on this effect, even a tomographic mapping capability of s-SNOM has been suggested [6,41]. Our present observation is the first report to distinguish different phonon resonances in both the covering layer and the buried material. We estimate from the observed amplitudes that the thickness of the "phosphate" particles is on the order of 10–30 nm, in agreement with their topographic appearance.

The origin of the "phosphate" particles remains unclear in this proof-of-principle study. Their erratic distribution may suggest some unknown preparation artifact. The material could be a modification of materials in the organic matrix; however, this is not highlighted in the infrared images. Nevertheless, it is clear that the particles could not simply be dried polishing material (Struers OP-A) since this shows a weak FTIR absorption at 1073 cm^−1^ (Figure 5d) but no discernible nano-FTIR resonance in the frequency range of interest. A strong argument for the assignment of the particles as crystalline phosphate is the observed high spectral phase effect of about 80°, exceeding that of bioaragonite (50°) and biocalcite (70°). Typically the spectral phase effect is on the order of 30° for strong polymer vibrations [8–9] but on the order of 400° for strong crystal phonons [3,6]. For molluscs the employment of phosphate in shell architecture has not been reported, but the radula (tooth structure) of the chitons is known to contain calcium phosphate [42–43]. In bones, phosphorylated proteins have been suggested as important components of the organic matrix [44–45]. Notwithstanding their unclear origin, our finding of "phosphate" particles demonstrates that nano-FTIR can easily locate and chemically recognize nanometer-sized material even at high rarefaction. We finally note that the observed particles are crystalline for two more reasons: (i) Their near-field scattering amplitude is about 10^−3^ as with calcite (Figure 3b and Figure 4), and not much smaller than 3 × 10^−3^ as known for two strongly polar crystals, SiC and SiO_2_ [3]; and (ii) their near-field resonance line shape is asymmetric, with the steep high-frequency edge (Figure 4) typical of strong oscillators [6,46]. Disorder in a crystal would strongly reduce the amplitude, as has been shown systematically [47]. Amorphous materials have a reduced, broadened resonance [3], while typical organic materials are known to have an even weaker response [8], as is also seen in this study with the PMMA resonance peaking at 1.5 × 10^−4^ near 1150 cm^−1^ (grey curve in Figure 8).

**Figure 12:**
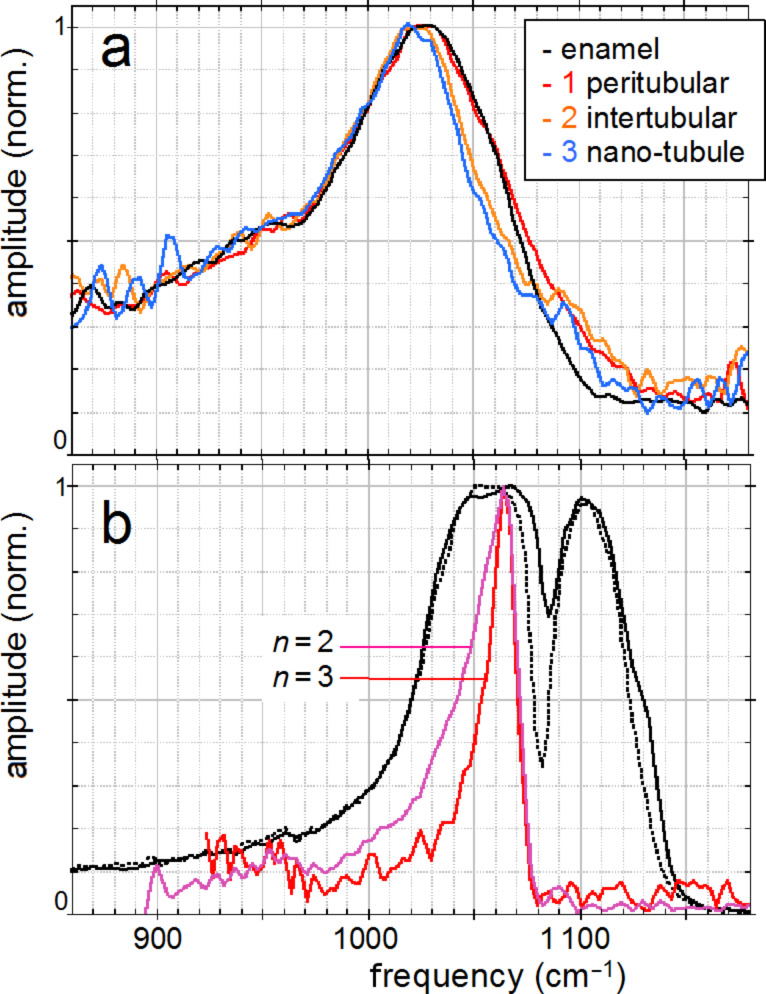
Normalized infrared resonance of phosphate; (a) of dentin and enamel measured by nano-FTIR (colors as in Figure 8); (b) of fluorapatite crystal at 10 nm tapping amplitude by nano-FTIR (magenta), and measured simultaneously at *n* = 3 demodulation (red, see Experimental section), and by conventional FTIR reflectance (black); the latter also for hydroxyapatite (black, dotted).

The broad phosphate bands measured in dentin by nano-FTIR contain information on the biomineral composition and density. Firstly, from their peak and baseline amplitudes (Figure 8) we tentatively determine the local volume fraction *f* of mineral particles (assuming *f* = 1 for enamel) to amount to *f* = 0.54, 0.30, and 0.26, respectively, for the spectra 1, 2, and 3 (see Experimental section). Then, following normalization, we obtain the line shapes of the mineral fraction at each of the three locations, plotted in Figure 12a (in the same colors as in Figure 8). Clearly there are significant, position-dependent differences in the 1020 to 1120 cm^−1^ frequency range. These differences show that (i) tooth materials consist, even on a 20 nm length scale, of several mineral types differing in their vibrational resonances, and (ii) the mineral composition varies with location. Specific spectral components may be identified at 1020, 1055, and 1100 cm^−1^. The component at 1055 cm^−1^ is present in enamel and peritubular dentin but not in intertubular dentin, and may relate to the lack of collagen protein, whereas the component at 1100 cm^−1^ is present in all dentin but not in enamel. For discussing possible assignments we have calculated and plotted the distribution of three characteristic quantities, which we extract from the nano-FTIR spectral scans, namely the peak s-SNOM amplitude (red), the ratio *r* of amplitudes at 1053 cm^−1^ and 1022 cm^−1^ (green; not meaningful in the tubule lumen), and the phase at 1080 cm^−1^ (blue), in a direct comparison with the BEI profiles (Figure 9 and Figure 10).

In the BE image with 0.12 nm resolution (Figure 7c), the brightness provides a local measure of the electron density [48] and consequently of the mineral content [39]. While the white hypermineralized rim of the tubule exhibits an identical shape to that seen in the infrared (Figure 7b), the fine linear fibrils in the BEI are not seen in the infrared images presumably because they are too deep below the surface. We note in passing that s-SNOM is nondestructive, unlike SEM in which the interaction of electrons with bony materials is known to induce damage. The extracted BE profiles (Figure 9) mark the edges of the tubule lumen, as do the extracted infrared profiles. Outside the peritubular rim, the amplitude (red) and phase (blue) correlate qualitatively with the BE-defined mineral content (black). An exception in the 7.1–7.5 µm section of Figure 9 (right) is attributed to the different probing depths of the s-SNOM and BE imaging methods. Amplitude and phase thus appear to be equally capable of measuring small density changes. As for the spectral differences within the phosphate band, the ratio *r* is about 0.8 and 0.7 for the peritubular and intertubular regions of the large tubule (x,y), respectively, but interestingly only 0.7 and 0.6, respectively, for the small tubule (z, Figure 10). For enamel *r* = 0.80 (Figure 12a).

An assignment of the observed nano-FTIR spectral components of tooth at around 1020, 1055, and 1100 cm^−1^ is, unfortunately, not straightforward, because most apatite species of interest have not yet been measured by s-SNOM as pure substances. For bulk crystals, it is well known from theory and experiments that the near-field resonance in the case of a strong oscillator is up-shifted from the transverse phonon frequency that marks the infrared absorption [6]. The up-shift nearly to the longitudinal phonon frequency amounts to 62 cm^−1^ for SiO_2_ [3], and even to 120 cm^−1^ for the exceptionally strong phonon of SiC [46]. For fluorapatite, infrared-active modes are known to be at 1030 cm^−1^ (strong), 1042.5 cm^−1^ (weak), and 1091 cm^−1^ (medium) [49], while nano-FTIR registers a strong resonance at 1063 cm^−1^ (as also in hydroxyapatite) and a weak one at 1090 cm^−1^, as shown in Figure 12b (for comparison we also show reflectivity spectra that nearly match for both apatites). The strong near-field resonance obviously comes from the strong infrared-active mode at 1030 cm^−1^, and thus is up-shifted by 33 cm^−1^. Naively one would expect that the near-field components observed at 1020, 1055, and 1100 cm^−1^ in tooth materials connect to correspondingly lower-frequency, strong infrared absorption components. But this seems not to be the case, because the experimental FTIR absorption of dentin exhibits peaks at 1039, 1069, 1108 [30], or 1040, 1060, 1092 cm^−1^ [28]. A down-shift of the 1040 line to 1014 cm^−1^ was reported for caries-affected dentin [26]. Theoretically it has not been explored for the case of small particles as to whether, and in which direction, the near-field resonance should shift from a given far-field absorption peak. Our experiments show that the near-field resonance in enamel and dentin exhibits a peak near 1020 cm^−1^, which is 43 cm^−1^ below the near-field resonance of apatite (Figure 12).

Generally, the interpretation of infrared absorption observed in bone should be extended to include the influence of the particles' shape through depolarization effects [50–51]. Recently, density functional theory has been applied specifically to the apatite ν_3_ vibrational infrared absorption, predicting strong spectral distortion and splitting (up to ±50 cm^−1^) due to these macroscopic electrostatic effects (not to be confused with microscopic distortion of lattice cells), depending on whether the particles are spherical, needle-like or plate-like [52]. Powder measurements with classical FTIR displayed absorption peaks at 1038, 1067, 1097 cm^−1^ for fluorapatite, and at 1034, 1053, 1105 cm^−1^ for hydroxyapatite, where indeed the last two peaks were found to be strongly split by the nonspherical shape of the particles [52]. Similar values were reported in other studies [24,53–54]. As the mineral in dentin and bone consists of isolated, locally ordered apatite platelets, strong depolarization effects probably distort the infrared spectra in the ν_3_ phosphate resonance region. Clearly a systematic study is warranted in which near-field and far-field infrared apatite bands are acquired for various shapes of chemically and structurally well-defined nanocrystals. Such a study should also cover the weaker ν_1_ phosphate band, which is less affected by electrostatic effects, as are all Raman lines [52].

Figure 12b directly illustrates the spectral discrimination provided by nano-FTIR [46], which has great potential for mineral research. The measured near-field response is seen to drop within 7 cm^−1^ (between 90% and 10% of the peak amplitude); the response is even sharper because our present instrumental resolution is about 6 cm^−1^ [3]. Additionally, Figure 12b shows that the resonance becomes narrowed simply by choosing a higher order *n* of signal demodulation (see Experimental section) [6]. This would result in a virtual "tip sharpening" and improve the spatial resolution of the s-SNOM [6,55–56]. As for the spectral resolution, a discrimination of components differing by just a few cm^−1^ is certainly achievable.

## Experimental

### s-SNOM near-field microscope

We employed a commercial scattering near-field microscope based on AFM (NeaSNOM, neaspec.com) equipped with a standard metalized tip (NCPt arrow, nanoandmore.com). It is operated in AFM tapping mode to modulate the near-field interaction between the tip and sample, and records the backscattered infrared signal simultaneously with the topography. Typical tapping amplitudes are 50–60 nm. Lock-in detection at the *n* = 2 harmonic (default) of the tapping frequency (approx. 300 kHz) provides background-free near-field imaging. Monitoring of the infrared signal versus tip–sample separation (approach curves) was used to ensure the optimal working settings of the tapping amplitude, the demodulation order *n*, and the focusing. In the monochromatic infrared near-field imaging mode of the s-SNOM a line-tunable CO_2_ laser attenuated to 10 mW is used for illumination. The acquisition time was 5 ms per pixel, requiring several minutes for a 128 × 128 sized image.

### Nano-FTIR mode of s-SNOM

The nano-FTIR spectroscopic mode of s-SNOM uses illumination by a coherent broadband mid-infrared beam (here 25 µW) from a difference-frequency source [3] driven by a femtosecond (<100 fs) Er fiber laser (FFS.SYS-2B and FFS-CONT, toptica.com). Detection and spectral analysis of the backscattered light is by an asymmetric Michelson interferometer that generates, by online Fourier transformation, infrared amplitude and phase spectra simultaneously; a switchable reference path ensures an absolute quantification of backscattering [3]. Note that while common FTIR spectrometers are not equipped to determine the complete, complex material response, the nano-FTIR phase spectra valuably complement the amplitude spectra [6]. For example, the phase change on resonance can be taken as a measure of the resonance strength. Nano-FTIR spectra can be monitored in real time at 3 Hz rate allowing the optimal focus adjustment on the tip. The usual acquisition time was 10 s per pixel for obtaining highly resolved spectra as in Figure 3, Figure 5 and Figure 8. The spectroscopic line scans in Figure 9 and Figure 10b were obtained with a reduced spectral resolution of about 8 cm^−1^, and the shown result is an average over five consecutive scans.

Figure 12b illustrates that the use of the *n* = 3 instead of the *n* = 2 demodulation order reduces the apatite resonance halfwidth by 40%. However, this is paid for by a five-fold reduction of the amplitude, as noted with other crystals previously [3]. Higher power than the presently available 25 µW would certainly allow for routine use of *n* = 3 and higher. Up to 10 mW is desirable (at which point tip heating starts to reduce the AFM stability) and would thus increase the present signal levels by 400×, or alternatively, reduce the acquisition time by 160,000× for a constant S/N ratio. Note that this positive perspective is in sharp contrast to tip-enhanced Raman scattering (TERS) for which up to 10 mW is readily available, but intrinsically weak cross sections leave little room for future signal improvement [57].

A spectrally integrated mode of nano-FTIR is also introduced in this study. It employs a fixed interferometer setting at a (free-induction-decay) [2] fringe maximum (ca. 150–300 fs delay). The detector amplitude signal then represents the background-suppressed near-field signal response averaged over a wide spectral band around the peak of the backscattered spectrum. Again the routine scanning is at a rate of 5 ms per pixel, requiring several minutes for a 128 × 128 pixel image.

### Sample preparation

The shell valve of *M. edulis* was sectioned longitudinally into 200 µm thick wafers. These were polished on both sides and etched for 45 s with a suspension of alumina nanoparticles (Struers OP-A), then cleaned and dried. Tooth samples were embedded in PMMA following dehydration by a graded ethanol and PMMA exchange solution; samples were cut perpendicularly to the tubules, serially ground and polished by using diamond slurry down to 1 µm [58].

### Line-shape determination of mineral component in a composite

A theory of near-field interaction for the dentin and bone cases of mixed particles that are smaller than the tip radius is not yet available. A straightforward solution would be to calculate an effective dielectric function, by using composite-medium theory [59], as a weighted average of the dielectric functions of the individual components (and taking proper account of depolarization), and then to apply the point-dipole or, better, the finite-dipole model of near-field interaction [3]. While composite-medium theory traditionally assumes spherical particles, an extension to ellipsoids is available [60]. Since individual dielectric functions are not known, however, we attempt here, for the first time in near-field microscopy, a simplified two-component analysis to extract the spectral contribution due to minerals. First, we determine the volume fraction *f* of mineral nanoparticles by extracting *f* from the spectra in Figure 8 in the following way. We assume the total scattering amplitude *s* to be a weighted sum of a mineral and an organic part, *s*^M^ and *s*^O^, respectively, *s* = *f s*^M^ + (1 − *f*) *s*^O^. We assume *f* = 1 for enamel, which consists nearly entirely of hydroxyapatite nanocrystals. For simplicity we assume a flat spectrum *s*^O^. By setting *s*^O^ = 0.00006 we then determine *f* = 0.54, 0.30, and 0.26 for the spectra 1, 2, and 3, respectively. With these values we compute the mineral component normalized amplitude spectra, *s*^M^ = (1 − (1 − *f*) *s*^O^/*s*)/*f* shown in Figure 12a. Other settings of *s*^O^ would give less agreement of the spectra outside the phosphate band.

## Conclusion

We have quite generally demonstrated the achievement of chemical identification—a central need for nanoscience—by an infrared nanoscope, at 20 nm resolution. We show both the highlighting of a selected compound in a scanned image, as well the measurement of local FTIR spectra. Our method is nondestructive and needs no vacuum or special sample preparation. Nano-FTIR is widely valuable for studying promising nanostructures, be it in nanotechnology, the pharmaceutical industry, or solid-state physics. For this study we have chosen biominerals over other obvious candidates because biomineralization is unexplored in its nanometer-scale detail but is yet of great medical importance.

## Author contributions

F. K. conceived this study, P. Z., W. W. S. and E. G. identified and characterized the biomineral samples. F. K. and S. A. designed the SNOM experiments and analyzed the results. S. A., P. Z. and Y. K. performed the experiments. F. K. wrote the draft, and all authors contributed to the manuscript.

## References

[1] Griffiths P R, de Haseth J A (2007). Fourier Transform Infrared Spectroscopy.

[2] Amarie S, Ganz T, Keilmann F (2009). Opt Express.

[3] Amarie S, Keilmann F (2011). Phys Rev B.

[4] Huth F, Schnell M, Wittborn J, Ocelic N, Hillenbrand R (2011). Nat Mater.

[5] Knoll B, Keilmann F (1999). Nature.

[6] Keilmann F, Hillenbrand R, Richards D, Zayats A (2009). Nano-Optics and Near-Field Optical Microscopy.

[7] Cvitkovic A, Ocelic N, Hillenbrand R (2007). Nano Lett.

[8] Taubner T, Hillenbrand R, Keilmann F (2004). Appl Phys Lett.

[9] Brehm M, Taubner T, Hillenbrand R, Keilmann F (2006). Nano Lett.

[10] Kim Z H, Liu B, Leone S R (2005). J Phys Chem B.

[11] Huber A J, Keilmann F, Wittborn J, Aizpurua J, Hillenbrand R (2008). Nano Lett.

[12] Qazilbash M M, Brehm M, Chae B-G, Ho P-C, Andreev G O, Kim B-J, Yun S J, Balatsky A V, Maple M B, Keilmann F (2007). Science.

[13] Hillenbrand R, Keilmann F (2002). Appl Phys Lett.

[14] Hillenbrand R, Keilmann F (2000). Phys Rev Lett.

[15] Lowenstam H A (1981). Science.

[16] Weiner S, Wagner H D (1998). Annu Rev Mater Sci.

[17] Meldrum F C, Cölfen H (2008). Chem Rev.

[18] Schmahl W W, Griesshaber E, Neuser R, Lenze A, Job R, Brand U (2004). Eur J Mineral.

[19] Griesshaber E, Schmahl W W, Neuser R, Pettke T, Blüm M, Mutterlose J, Brand U (2007). Am Mineral.

[20] Goetz A J, Steinmetz D R, Griesshaber E, Zaeffere S, Raabe D, Kelm K, Irsen S, Sehrbock A, Schmahl W W (2011). Acta Biomater.

[21] Merkel C, Griesshaber E, Kelm K, Neuser R, Jordan G, Logan A, Mader W, Schmahl W W (2007). J Geophys Res.

[22] Schmahl W W, Griesshaber E, Merkel C, Kelm K, Deuschle J, Neuser R D, Göetz A J, Sehrbrock A, Mader W (2008). Mineral Mag.

[23] Rey C, Shimizu M, Collins B, Glimcher M J (1991). Calcif Tissue Int.

[24] Pleshko N, Boskey A, Mendelsohn R (1991). Biophys J.

[25] Carden A, Morris M D (2000). J Biomed Opt.

[26] Spencer P, Wang Y, Katz J L, Misra A (2005). J Biomed Opt.

[27] Boskey A, Mendelsohn R (2005). J Biomed Opt.

[28] Abraham J A, Sánchez H J, Marceli C A, Grenón M, Guidi M C, Piccinini M (2011). Anal Bioanal Chem.

[29] Paschalis E P, Mendelsohn R, Boskey A L (2011). Clin Orthop Relat Res.

[30] Tesch W, Eidelman N, Roschger P, Goldenberg F, Klaushofer K, Fratzl P (2001). Calcif Tissue Int.

[31] Paschalis E P, DiCarlo E, Betts F, Sherman P, Mendelsohn R, Boskey A L (1996). Calcif Tissue Int.

[32] Gourion-Arsiquaud S, Faibish D, Myers E, Spevak L, Compston J, Hodsman A, Shane E, Recker R R, Boskey E R, Boskey A L (2009). J Bone Miner Res.

[33] Dalbeck P, England J, Cusack M, Lee M R, Fallick A E (2006). Eur J Mineral.

[34] Feng Q L, Li H B, Pu G, Zhang D M, Cui F Z, Li H D, Kim T N (2000). J Mater Sci.

[R35] 35Griesshaber, E.; Kelm, K.; Jordan, G.; Xu, D.; Schmahl, W. W. In preparation.

[36] Taubner T, Keilmann F, Hillenbrand R (2005). Opt Express.

[37] Zaslansky P, Fratzl P (2008). Collagen: Structure and Mechanics.

[38] Mjör I A, Nordahl I (1996). Arch Oral Biol.

[39] Roschger P, Fratzl P, Eschberger J, Klaushofer K (1998). Bone.

[40] Raschke M B, Lienau C (2003). Appl Phys Lett.

[41] Sun J, Schotland J C, Hillenbrand R, Carney P S (2009). Appl Phys Lett.

[42] Lowenstam H A, Weiner S (1985). Science.

[43] Lee A P, Brooker L R, Macey D J, van Bronswijk W, Webb J (2000). Calcif Tissue Int.

[44] Thurner P J, Lam S, Weaver J C, Morse D E, Hansma P K (2009). J Adhes.

[45] Becker A, Ziegler A, Epple M (2005). Dalton Trans.

[46] Hillenbrand R, Taubner T, Keilmann F (2002). Nature.

[47] Ocelic N, Hillenbrand R (2004). Nat Mater.

[48] Wells O C (1977). Scanning Electron Microsc.

[49] Kravitz L C, Kingsley J D, Elkin E L (1968). J Chem Phys.

[50] Fuchs R (1975). Phys Rev B.

[51] Bohren C F, Huffmann D R (1983). Absorption and Scattering of Light by Small Particles.

[52] Balan E, Delattre S, Roche D, Segalen L, Morin G, Guillaumet M, Blanchard M, Lazzeri M, Brouder C, Salje E K H (2011). Phys Chem Miner.

[53] Penel G, Leroy G, Rey C, Sombret B, Huvenne J P, Bres E (1997). J Mater Sci: Mater Med.

[54] Leroy G, Leroy N, Penel G, Rey C, Lafforgue P, Bres E (2000). Appl Spectrosc.

[55] Knoll B, Keilmann F (2000). Opt Commun.

[56] Giessibl F J (1995). Science.

[57] Richter M, Hedegaard M, Deckert-Gaudig T, Lampen P, Deckert V (2011). Small.

[58] Zaslansky P, Zabler S, Fratzl P (2010). Dent Mater.

[59] Bruggeman D A G (1935). Ann Phys (Berlin, Ger).

[60] Hinrichs K, Röseler A, Roodenko K, Rappich J (2008). Appl Spectrosc.

